# Characterization of quinolone-resistant Enterobacteriaceae strains isolated from poultry in Western Algeria: First report of *qnrS* in an *Enterobacter cloacae*

**DOI:** 10.14202/vetworld.2018.469-473

**Published:** 2018-04-12

**Authors:** Qada Benameur, Hassiba Tali-Maamar, Farida Assaous, Badia Guettou, Meki Boutaiba Benklaouz, Kheira Rahal, Meriem-Hind Ben-Mahdi

**Affiliations:** 1 Faculty of Natural Sciences and Life, Abdelhamid Ibn Badis University, Mostaganem, Algeria; 2 Research Laboratory, Health and Animal Production, Higher National Veterinary School, Algiers, Algeria; 3 Medical Bacteriology Laboratory, Pasteur Institute of Algeria, Algiers, Algeria; 4 Veterinary Sciences Institute, Ibn Khaldoun University, Tiaret, Algeria; 5 Higher School of Food Sciences and Agro-alimentary Industries, Algiers, Algeria

**Keywords:** Algeria, antimicrobial resistance, Enterobacteriaceae, *qnrS1*

## Abstract

**Aim:**

Multidrug-resistant (MDR) Enterobacteriaceae have frequently been reported, in both human and veterinary medicine, from different parts of the world as a consequence of antibiotic usage. However, there is a lack of published data regarding antimicrobial resistance in non-*Escherichia coli* (*E. coli*) Enterobacteriaceae from animals in Algeria. This study aimed to evaluate the frequency of resistance to antibiotics with a focus on quinolones and to investigate the presence of *qnr* genes inEnterobacteriaceaeof poultry origin.

**Materials and Methods:**

A total of 310 samples of poultry origin were collected from 2010 to 2014 from broiler and layer farms and hatcheries located in different geographic areas of Western Algeria (including Mostaganem, Oran, Mascara, Relizane, Chlef, Tiaret, and Tissemsilt). Antimicrobial susceptibility testing was performed using disc diffusion assay. Polymerase chain reaction and sequencing accomplished the characterization of *qnr* genes (*qnrA*, *qnrB*, and *qnrS*).

**Results:**

A total of 253 Enterobacteriaceaestrains were isolated in this study. These isolates exhibited high levels of resistance to quinolones and other families of antibiotics. All the strains isolated in this study were resistant to at least one antibiotic. Among them, 233 (92.09%) were considered MDR. Among the 18 randomly selected nalidixic acid (NA)-resistant Enterobacteriaceaeisolates, one *E. coli* and one *Enterobacter cloacae* were carrying *qnrS1*. By contrast, *qnrA* and *qnrB* were not detected in this study.

**Conclusion:**

This is the first report on the identification of the *qnrS* gene in *E. cloacae* isolated from animal source in Algeria. Further studies have to be conducted to determine the real prevalence of *qnr* genes.

## Introduction

Quinolones and fluoroquinolones are broad-spectrum antimicrobial agents, extensively used in poultry disease treatment. This widespread use has been associated worldwide with an increased level of resistance, especially in Gram-negative bacteria species in the last decade [1,2]. Multidrug-resistant (MDR) Enterobacteriaceae have frequently been reported, in both human and veterinary medicine, from different parts of the world as a consequence of antibiotic usage [3,4]. In Algeria, the frequency of antimicrobial resistance in *Escherichia coli* (*E. coli*)from animals has already been reported by several authors [5-8]. However, there is a lack of published data regarding antimicrobial resistance in non-*E. coli* Enterobacteriaceaefrom animals in Algeria. It is admitted that resistance to quinolones results from both chromosomal and plasmid-mediated quinolone resistance (PMQR) mechanisms. *Qnr* genes represent one of the most important PMQR mechanisms. These genes encode pentapeptide repeat proteins that block the action of ciprofloxacin (CIP) on bacterial DNA gyrase and topoisomerase IV [9,10]. Three major groups of Qnr determinants have been described (QnrA, QnrB, and QnrS), which share between 40% and 60% similarity [11]. These determinants have been identified worldwide in various Enterobacteriaceae,and they have frequently been associated with extended-spectrum β-lactamases (ESBLs) and plasmidic cephalosporinases [12,13].

In Algeria, these determinants have been reported in various human Enterobacteriaceae [14-16]. However, the occurrence of these resistance determinants in isolates of animal origin in Algeria is rarely documented.

This study aimed to evaluate the frequency of resistance to quinolones and other groups of antibiotics in Enterobacteriaceae isolated from poultry in Western Algeria and to investigate the presence of *qnr* genes in a collection of nalidixic acid (NA)-resistant Enterobacteriaceaeisolates.

## Materials and Methods

### Ethical approval

Ethical approval is not needed to pursue this type of study. However, no chickens were harmed during the collection of samples.

### Bacterial strains

From December 2010 to January 2014, 253 non-duplicate Enterobacteriaceae strains were isolated from 310 samples received in the Regional Veterinary Laboratory of Mostaganem, Algeria, for routinely control of *Salmonella*. All samples were collected by veterinarians controlling from farms and hatcheries located in different geographic areas of Western Algeria (including Mostaganem, Oran, Mascara, Relizane, Chlef, Tiaret, and Tissemsilt). The samples nature received were healthy and diseased broiler and layer breeders, 1 day-old broiler and layer chicks, broilers, laying hens, and farm swabs. The isolates were recovered from internal organs (liver, spleen, or pericardium), fecal samples, or farm swabs. For the primary isolation, one mL of sample was inoculated with nine mL of buffered peptone water vortexed and incubated at 37°C overnight. Then, a drop of broth was streaked on MacConkey agar and incubated at 37°C overnight. The Enterobacteriaceae isolates were identified biochemically by classical biochemical testing or using the API 20E system (bioMérieux, Marcy l’Étoile, France).

### Antimicrobial susceptibility testing

The antimicrobial susceptibility of all isolated Enterobacteriaceae strains was tested following Clinical and Laboratory Standards Institute (CLSI) guidelines [17]. The isolates were tested against a panel of 12 antimicrobials: Nalidixic acid (NA, 30 µg), flumequin (UB, 30 µg), ciprofloxacin (CIP, 5 µg), ampicillin (AM, 10 µg), amoxicillin/clavulanic acid (AMC, 20/10 µg), ceftiofur (XNL, 30 µg), tetracycline (TE, 30 µg), trimethoprim/sulfamethoxazole (SXT, 1.25/23.75 µg), neomycin (N, 30 µg), gentamicin (CN, 15 µg), chloramphenicol (C, 30 µg), and colistin (CT, 50 µg) (Bio-Rad, Marnes la Coquette, France). Results were obtained after incubating samples for 16–18 h at 37°C and were interpreted according to CLSI previously cited guidelines. *E. coli* ATCC 25922 was used as a quality control strain.

### Polymerase chain reaction (PCR) and DNA sequencing

A total of 18 MDR isolates randomly selected among NA-resistant Enterobacteriaceae isolates werescreened by multiplex PCR amplification of *qnrA, qnrB*, and *qnrS* as previously described [18-20], after extraction of total DNA by the boiling method. Primers used were as follows: for *qnrA*, 5’-TTCTCACGCCAGGATTTGAG and 5’-TGCCAGGCACAGATCTTGAC, to give a 571 pb product; for *qnrB*, 5’-TGGCGAAAAATT(GA)ACAGAA and 5’-GAGCAACAG(TC)GCCTGGTAG, to give a 594 pb product; and for *qnrS*, 5’-GACGTGCTAACTTGCGTGAT and 5’-GACGTGCTAACTTGCGTGAT, to give a 388 pb product. All the six primers were added to the template DNA and PCR mix (Invitrogen, Carlsbad, CA). The following cycle conditions were used: Initial denaturation at 95°C for 5 min, followed by 30 cycles of denaturation at 94°C for 1 min, annealing at 60°C for 45 s and amplification at 72°C for 1 min, and a final extension at 72°C for 10 min.Negative controls (without DNA template) were included in each run. Amplification products were provisionally identified from their sizes in agarose gels. Amplification products were separated by electrophoresis, on 1.5% ethidium bromide-stained agarose gels in 1 × TBE buffer at 150 V for 1 h, and then visualized under ultraviolet light. PCR amplicons were confirmed by sequencing and the DNA sequences obtained were compared with those in the GenBank using the BLAST program (http://www.ncbi.nlm.nih.gov/BLAST).

## Results and Discussion

### Antimicrobial susceptibility of Enterobacteriaceae isolates

**Figure-1:**
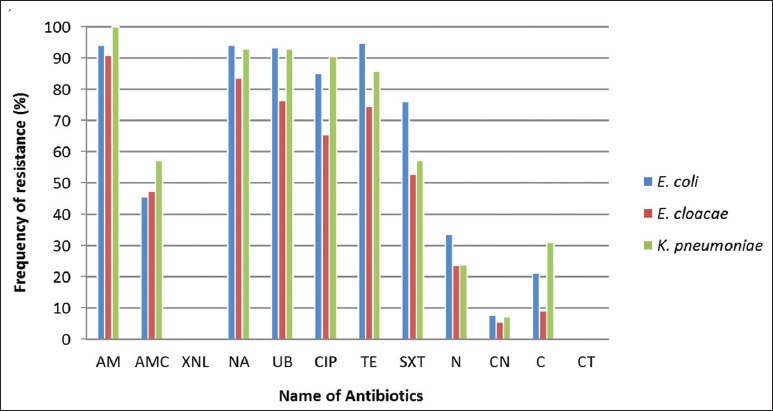
Antimicrobial resistance among Enterobacteriaceae. AM=Ampicillin, AMC=Amoxicillin/clavulanic acid, XNL=Ceftiofur, NA=Nalidixic acid, UB=Flumequin, CIP=Ciprofloxacin, TE=Tetracycline, SXT=Trimethoprim/sulfamethoxazole, N=Neomycin, CN=Gentamicin, C=Chloramphenicol, CT=Colistin.

Two hundred and fifty three Enterobacteriaceae strains were isolated from 310 poultry samples received in the Regional Veterinary Laboratory of Mostaganem, Northwestern Algeria. The isolates consisted of 134 *E. coli*, 55 *Enterobacter cloacae (E. cloacae)*, 42 *Klebsiella pneumoniae (K. pneumoniae)*,10 *Proteus mirabilis*,7 *Serratia marcescens*,and 5 *Providencia rettgeri*. The percentage of antimicrobial resistance of the predominant Enterobacteriaceaestrains isolated in this study is shown in the Figure-1. *E. coli* isolates showed a high resistance rate to particular antimicrobials, notably TE 94.77% (n=127), NA 94.03% (n=126), AM 94.03% (n=126), UB 93.28% (n=125), CIP 85.10% (n=114), and SXT 76.11% (n=102). Among *E. cloacae* isolates, the highest proportion of resistance was toward AM 90.90% (n=50), followed by NA 83.63% (n =46), UB 76.36% (n=42), TE 74.54% (n=41), CIP 65.45% (n=36), and SXT 52.72% (n=29). Resistance of *K. pneumoniae* to AM, NA, UB, CIP, TE, SXT, and AMC was, respectively, observed in 100% (n=42), 92.85% (n=39), 92.85% (n=39), 90.47 % (n=38), 85.71% (n=36), 57.14 % (n=24), and 57.14% (n=24) of the isolates. All the isolates examined in this study were resistant to at least one antibiotic. Among them, 233 (92.09%) were considered MDR (resistant to three or more different antimicrobial agents belonging to different classes of antibiotics) (Table-1). *K. pneumoniae* and *E. coli* are the most common opportunistic Enterobacteriaceae, and their growing cross-resistance to quinolones is a critical problem [21,22]. In Algeria, there is a lack of published data regarding antimicrobial resistance in non-*E. coli* and *non-Salmonella* spp. Enterobacteriaceae of animal origin. Resistance of *E. coli* isolatesto quinolones was far higher compared to previous studies conducted in Algeria [6,7]. In view of the whole range of antibiotics available in Algeria and the increasing and inappropriate use of quinolones in poultry farms, the globally high incidence of antibiotic resistance observed in this study is not really surprising.

**Table-1:** MDR in Enterobacteriaceae isolates.

Organism	Total No.	Number of MDR isolates (%)
*E.coli*	134	125 (93.28)
*E. cloacae*	55	52 (94.54)
*K. pneumoniae*	42	39 (92.85)
*P. mirabilis*	10	8 (80.00)
*S. marcescens*	7	5 (71.42)
*P. rettgeri*	5	4 (80.00)
Total	253	233 (92.09)

MDR=Multidrug resistant, *E.coli=Escherichia coli,E. cloacae=Enterobacter cloacae,K. pneumoniae=Klebsiella pneumoniae,P. mirabilis=Proteus mirabilis,S. marcescens=Serratia marcescens,* *P. rettgeri=Providencia rettgeri*

### Qnr occurrence

The *qnr* multiplex PCR allowed the detection of two positive isolates (one *E. coli* and one *E. cloacae*), both of them were carrying *qnrS1* (Table-2), whereas *qnrA* and *qnrB* were not identified in any of the 18 randomly selected isolates. In Algeria, several studies allowed the detection of *qnr* determinants in human clinical isolates [12-14]. However, few studies reported their presence in isolates of animal origin. *qnrA* has been recently identified in ESBL producing *E. coli* isolates from poultry [23], and *qnrS1* and *qnrB5* in ESBL producing *E. coli* isolates from companion animals [24]. To the best of our knowledge, this is the first description of *qnrS* genes in an *E. cloacae* isolate from animal source in Algeria. All of the previously cited Algerian studies detected the presence of *qnr* determinants in ESBL producing isolates. However, no study reported their presence in non-ESBL Enterobacteriaceae in our country. The *qnrS* gene has been previously detected in several *Salmonella* strainsisolated from poultry source in Denmark, Germany, and Netherland [25-27] and was also reported in *E. coli* isolates from food-producing animals in China and Nigeria [28,29]. Typically, *qnrB* was considered to be the most prevalent PMQR gene in Enterobacteriaceae isolates [11]. The other 16 NA -resistant Enterobacteriaceae isolates tested in this study were negative for the three *qnr* genes investigated. However, they were not tested for other *qnr* determinants (*qnrC, qnrD*, and *qnrVC*). Thus, this preliminary study have to be completed by further investigations of other PMQR determinants, including *qnrC,qnrD*, *qnrVC*, *aac(6’)-Ib-cr, qepA*, and *oqxAB. qnr* genes have been either detected alone or in association with ESBL genes in a range of bacterial species [30]. Recently, *qnrA* or *qnrS* determinants were identified in non-ESBL-positive isolates harboring *TEM-1* or *LAP-1* [31]. As previously reported, our results confirmed that the spread of these genes can be independent and not always associated with *bla*_ESBL_ genes [2,31].

**Table-2:** Enterobacteriaceae isolates tested by PCR for the determination of *qnr* determinants.

Strain	*qnr*	Antimicrobial resistance pattern
*E.coli* (S1)	/	NA, UB, CIP, AM, SXT
*E.coli* (S2)	/	NAL, UB, CIP, AM, TE
*E.coli* (S3)	/	NAL, UB, CIP, AM, SXT, TE
*E.coli* (S4)	/	NAL, UB, CIP, AM, TE
*E.coli* (S5)	/	NAL, UB, CIP
*E.coli* (S6)	*qnrS1*	NAL, UB, CIP, AM, SXT, TE
*E.coli* (S7)	/	NAL, UB, CIP, AM, SXT, TE, N
*E.coli* (S8)	/	NAL, UB, CIP, AM, SXT, TE
*P. rettgeri* (S9)	/	NAL, UB, CIP, AM, AMC, SXT, TE, CN, C
*E.coli* (S10)	/	NAL, UB, SXT, TE
*K. pneumoniae* (S11)	/	NAL, UB, CIP, AM, SXT, TE, C
*E. cloacae* (S12)	*qnrS1*	NAL, UB, AM, AMC, TE, SXT
*E.coli* (S13)	/	NAL, UB, AM, AMC, SXT, TE
*E.coli* (S14)	/	NAL, UB, AM, AMC, SXT, TE
*E.coli* (S15)	/	NAL, UB, CIP, AM, SXT, TE, N
*E.coli* (S16)	/	NAL, UB, CIP, AM, SXT, TE
*E.coli* (S17)	/	NAL, UB, CIP, AM, SXT, TE, N
*E.coli* (S18)	/	NAL, UB, CIP, AM, SXT, TE, CN

NA=Nalidixic acid; UB=flumequine; CIP=Ciprofloxacin; SXT=Trimethoprim/sulfamethoxazole; TE=Tetracycline; N=Neomycin; CN=Gentamicin; C=Chloramphenicol, *E.coli=Escherichia coli, E. cloacae=Enterobacter cloacae,K. pneumoniae=Klebsiella pneumoniae,P. rettgeri=Providencia rettgeri*

*E. coli* harboring *qnrS1* gene, detected in our study, was resistant to CIP, whereas the *qnrS1* positive *E. cloacae* were susceptible to CIP. Quinolone resistance has been described to be transmitted by plasmids carrying *qnr* genes [32], resulting in low-level quinolone resistance and it can facilitate the selection of quinolone-resistant mutants with higher-level resistance [33,34]. The transferability of CIP -resistant *E. coli* or mobile resistance determinants from chickens to humans has been indicated in several studies [35,36]. Since the zoonotic transfer of fluoroquinolone-resistant bacteria is of concern from a human health perspective, the reverse scenario - the transfer of fluoroquinolone-resistant bacteria from humans to animals - warrants equal consideration, which may be responsible for therapeutic failures in animals.

## Conclusions

This study revealed high levels of antimicrobial resistance to antibiotics with a focus on quinolones in Enterobacteriaceaeisolates. This is the first detection of *qnrS* in *E. cloacae* isolates from the animal in Algeria. The emergence of PMQR thus may contribute by several means to the rapid and deleterious increase in bacterial resistance to fluoroquinolones. These fluoroquinolone-resistant bacteria may be transferred from animals to humans and vice versa, increasing the risk of treatment failure. Therefore, implementation of more efficient preventive measures at all levels of broiler and layer industries is becoming mandatory.

## Authors’ Contributions

QB, FA, BG, and MBB carried out the main research works and analyzed the main data in the experiments. HT, KR, and MHB have supervised thelaboratory work and approved the final version of the manuscript. All authors read and approved the final manuscript.
